# Considerable interobserver variation in delineation of pancreatic cancer on 3DCT and 4DCT: a multi-institutional study

**DOI:** 10.1186/s13014-017-0777-0

**Published:** 2017-03-23

**Authors:** Eva Versteijne, Oliver J. Gurney-Champion, Astrid van der Horst, Eelco Lens, M. Willemijn Kolff, Jeroen Buijsen, Gati Ebrahimi, Karen J. Neelis, Coen R. N. Rasch, Jaap Stoker, Marcel van Herk, Arjan Bel, Geertjan van Tienhoven

**Affiliations:** 1 Department of Radiation Oncology, Academic Medical Center, University of Amsterdam, Meibergdreef 9, 1105 AZ Amsterdam, The Netherlands; 2 Department of Radiology, Academic Medical Center, University of Amsterdam, Meibergdreef 9, 1105 AZ Amsterdam, The Netherlands; 3 Department of Radiation Oncology, MAASTRO clinic, Doctor Tanslaan 12, 6229 ET Maastricht, The Netherlands; 4 Department of Radiation Oncology, Leiden University Medical Center, Leiden University, Postbus 9600, 2300 RC Leiden, The Netherlands; 5 Faculty of Biology, Medicine & Health, Division of Molecular & Clinical Cancer Sciences, University of Manchester and Christie NHS trust, Oxford Road Manchester, M13 9PL Manchester, United Kingdom

**Keywords:** Pancreatic cancer, Delineation, Interobserver variation

## Abstract

**Background:**

The delineation of pancreatic tumors on CT is challenging. In this study, we quantified the interobserver variation for pancreatic tumor delineation on 3DCT as well as on 4DCT.

**Methods:**

Eight observers (radiation oncologists) from six institutions delineated pancreatic tumors of four patients with (borderline) resectable pancreatic cancer. The study consisted of two stages. In the 3DCT-stage, the gross tumor volume (GTV) was delineated on a contrast-enhanced scan. In the 4DCT-stage, the internal GTV (iGTV) was delineated, accounting for the respiratory motion. We calculated the volumes of the (i)GTV, the overlap of the delineated volumes (expressed as generalized conformity index: CI_gen_), the local observer variation (local standard deviation: SD) and the overall observer variation (overall SD). We compared these results between GTVs and iGTVs. Additionally, observers were asked to fill out a questionnaire concerning the difficulty of the delineation and their experience in delineating pancreatic tumors.

**Results:**

The ratios of the largest to the smallest delineated GTV and iGTV within the same patient were 6.8 and 16.5, respectively. As the iGTV incorporates the GTV during all respiratory phases, the mean volumes of the iGTV (40.07 cm^3^) were larger than those of the GTV (29.91 cm^3^). For all patients, CI_gen_ was larger for the iGTV than for the GTV. The mean overall observer variation (root-mean-square of all local SDs over four patients) was 0.63 cm and 0.80 cm for GTV and iGTV, respectively. The largest local observer variations were seen close to biliary stents and suspicious pathological enlarged lymph nodes, as some observers included them and some did not. This variation was more pronounced for the iGTV than for the GTV. The observers rated the 3DCT-stage and 4DCT-stage equally difficult and treated on average three to four pancreatic cancer patients per year.

**Conclusions:**

A considerable interobserver variation in delineation of pancreatic tumors was observed. This variation was larger for 4D than for 3D delineation. The largest local observer variation was found around biliary stents and suspicious pathological enlarged lymph nodes.

**Electronic supplementary material:**

The online version of this article (doi:10.1186/s13014-017-0777-0) contains supplementary material, which is available to authorized users.

## Background

The aim of radiotherapy is delivering a high radiation dose to the tumor while minimizing the dose to organs at risk (OARs). For pancreatic tumors, this is challenging due to day-to-day position variation, respiratory motion, and uncertainties in delineation of the tumor [1–4].

The radiation oncologist can delineate the gross tumor volume (GTV) on a three-dimensional CT (3DCT). The GTV is expanded with a margin to account for microscopic extensions, resulting in the clinical target volume (CTV). For the remaining uncertainties, such as organ motion and set-up uncertainties, an additional margin is added to form the planning target volume (PTV). Nowadays, a four-dimensional CT (4DCT) scan is increasingly used to account for tumor motion during respiration [1, 2], for example combined with the internal target volume (ITV) [5] or mid-ventilation approach [6]. For pancreatic cancer patients treated at our department, we combine 4DCT with a modified ITV approach. In this approach, the radiation oncologist delineates the GTV on the average scan of the 4DCT and expands that on all respiratory phases of the 4DCT to generate an internal GTV (iGTV). A 5 mm margin is then added to define the internal CTV (iCTV). An additional PTV margin is added, to account for remaining set-up uncertainties. This PTV margin can be smaller compared with 3DCT delineation since respiratory motion uncertainty is accounted for in this 4D approach. In both the 3DCT and 4DCT approaches it is important that appropriate margin size is used as too small a margin leads to under-treatment of the target volume whereas too large a margin leads to unnecessarily high doses to the OARs. The CTV or iCTV to PTV margins currently used to account for the delineation uncertainties in pancreatic cancer are largely based on assumptions of these uncertainties. To investigate whether these assumptions are correct we performed a delineation study.

Previous delineation studies quantified the interobserver delineation uncertainties for several tumor sites [7–11]. These studies resulted in standardized delineation protocols for those organs. For pancreatic cancer, such a protocol is available in both postoperative setting and preoperative setting [12, 13]. In the study of Carvatta et al., standard criteria for CTV delineation of high risk elective lymph node areas in preoperative or definitive treatment with radiotherapy were developed [13]. Both guidelines were promoted and evaluated in a multicenter dummy-run, and showed an acceptable interobserver variation in delineation of these elective CTVs [14]. Only two other multi-institutional studies on the delineation of pancreatic tumors are available [15, 16]. All three studies show large interobserver variation in GTV delineation, with ratios of the largest to the smallest GTV volume of 6.8 [14], 9 [15] and 3 [16]. Two of these studies were quality control studies of a clinical trial [15, 16]. Those studies only used 3DCT and included 1–2 patients with locally advanced pancreatic cancer [15, 16]. The third was a delineation study which included two patients and only investigated the interobserver variation using 3DCT [14]. All of these studies reported limited quantitative information (i.e., standard deviations, SD and generalized conformity index, CI_gen_) [14–16].

The aim of this study was to quantify the interobserver variation for GTV (3DCT) and iGTV (4DCT) delineations. The study included four patients with (borderline) resectable pancreatic cancer, and eight radiation oncologists from six institutions.

## Methods

Radiation oncologists (observers) from all nine institutions participating in the PREOPANC trial were asked to participate in this delineation study. Eight observers from six institutions actually participated.

### Patients’ characteristics

The data of four patients with histologically proven (borderline) resectable pancreatic tumors were used and anonymized. All patients gave written informed consent for both the PREOPANC trial (EudraCT number 2012-003181-40) and MIPA (NCT01989000) study and were the first four patients that randomized for preoperative radiochemotherapy at the Academic Medical Center (AMC) within the PREOPANC trial [17]. Both studies were approved by the local medical ethics committee (PREOPANC: Erasmus Medical Center, Rotterdam; MIPA: AMC, Amsterdam) [17]. Preoperative radiochemotherapy consisted of 15 fractions of 2.4 Gy combined with gemcitabine 1000 mg/m^2^ once a week for three weeks, preceded and followed by a modified course of gemcitabine 1000 mg/m^2^, once a week for two weeks. Between the three cycles there was one week rest [17].

### CT scans

#### Diagnostic CT scan

All patients had a contrast-enhanced diagnostic CT scan in the referring hospital, which was considered to be of adequate diagnostic quality by abdominal radiologists from the AMC with extensive experience in pancreatic cancer. The scans included an axial scan in arterial contrast phase (on average 35 s after injection, all patients), venous contrast phase (on average 60 s after injection, patients 1,2 and 4) and/or a portal contrast phase (on average 240 s after injection, patients 1 and 4) with or without reconstructed coronal views. Two experienced radiologists from the AMC reported the studies. The radiology report of patient 2 described two suspicious loco regional lymph nodes; the radiology report of patient 4 described some enlarged lymph nodes, which were not further characterized.

#### Planning CT scan

The planning CT scans were obtained at the radiation oncology department of the AMC with a GE LightSpeed RT 16 scanner (General Electric Company, Waukesha, WI) using a standard acquisition protocol (slice thickness of 2.5 mm). Patients were scanned in treatment position: supine on a flat table top with arms raised above their heads.

First, a 3DCT scan was obtained during free breathing after intravenous Iodine contrast injection. During the same CT session, a few minutes after the 3DCT scan, a 4DCT scan was obtained. The patient’s breathing motion was monitored and synchronized to the CT acquisition by the respiratory gating system RPM (Real-Time Position Management, Varian Oncology Systems, Palo Alto, CA). For the 4DCT, images were captured during continuous respiration and divided into ten respiratory bins, resulting in ten image sets of the respiratory cycle. Also, a maximum intensity projection (MIP) and an average intensity projection (Ave-IP) were reconstructed from the ten phase scans. The planning CT scan was obtained during the first modified course of gemcitabine (mean eight days after the first administration of gemcitabine), and on average six weeks (46–62 days, mean 53 days) after the diagnostic CT. The 3DCT and 4DCT scans were registered to each other but not to the diagnostic CT scans.

#### Fiducial markers and biliary stents

All four patients had intratumoral fiducial markers, which were placed under the guidance of endoscopic ultrasound (EUS), for position verification during radiotherapy [18, 19]. Patients 1, 3 and 4 had a pancreatic head tumor and had received three intratumoral Visicoil fiducial markers (RadioMed, Barlett, TN). For patient 2, two Gold Anchor fiducial markers (Naslund Medical AB, Huddinge, Sweden) and one Visicoil fiducial marker had been placed, but mistakenly in the pancreas head instead of in the corpus tumor. Also, all patients had biliary drainage: patients 1–3 had fully covered metal biliary stents, patient 4 had external percutaneous biliary drainage. All markers, biliary stents and percutaneous biliary drainage had been placed after the diagnostic CT scans and were thus only visible on the planning CT scan.

### Delineation software

The Big Brother software, dedicated to radiotherapy delineation studies was used, recording delineations as well as observer-computer interactions [8]. Each observer received a USB stick containing all CT scans, the radiology report, the Big Brother software, and delineation instructions. These instructions were identical to those in the PREOPANC trial protocol [17].

### Delineation protocol

The study consisted of a 3DCT-stage and a 4DCT-stage.

In the 3DCT-stage, the observers were asked to delineate the GTV on the 3DCT scan, which was displayed on the main window. The GTV was defined as the macroscopically visible tumor and neighboring suspicious pathological lymph nodes. A separate window was available for viewing the diagnostic CT scans. A margin of 5 mm was automatically applied to create the CTV.

In the 4DCT-stage, the Ave-IP reconstruction was displayed in the main window. The observers were asked to delineate the GTV on the Ave-IP reconstruction and then create an iGTV defined as the volume encompassing the GTV on all ten respiratory phase image sets of the 4DCT. The diagnostic CT scan, 3DCT scan, and remaining 4DCT images including the MIP reconstruction were available in a separate window. As the 3DCT and 4DCT scans from the planning CT were obtained in the same session, the 3DCT and 4DCT scans were linked to the Ave-IP reconstruction displayed in the main window. Furthermore, a copy of the cursor (linked cursor) was displayed at the corresponding location in the secondary window when these scans were displayed. Once finished with the iGTV delineations, a margin of 5 mm was automatically applied to create the iCTV. Completed delineations were sent back to the investigators by email.

### Questionnaire

Observers were asked to fill out a questionnaire containing eight questions about the delineation process (Additional file 1). These multiple choice questions about the delineation process included answers ranging from very easy to very difficult in five steps. In addition, there were three questions about the experience of the observer in delineating pancreatic tumors as well as the number of pancreatic cancer patients the observers treated yearly within and outside the PREOPANC study (Additional file 1).

### Data analysis

The data were analyzed using the Big Brother software [8]. The following analyses were repeated for the GTV, iGTV, CTV and iCTV data.

Scatterplots were generated in GraphPad Prism (version 5.00, GraphPad Software, San Diego, CA) to present the range of delineated volumes. Using the Big Brother software we calculated the average volume of the (i)GTV and CI_gen_ for each patient [20]. The CI_gen_ is a measure of overlap of the delineated volumes and is defined as the ratio of the sum over all observer pairs of the volumes common to both observers and the sum over all observer pairs of the encompassing volumes (volume delineated by at least one of the two observers) [20]. CI_gen_ ranges from 0–1, where 1 indicates full overlap of the delineated volumes from all observers and 0 indicates no overlap. To assess the accuracy of CI_gen_ we repeated its calculation a number of times equal to the number of observers, leaving out one different observer at each repetition. The range of results from this leave-one-out procedure was reported. To test for significant differences in average volumes, we used a two-sided Wilcoxon signed-rank test (32 pairs, significance level α = 0.05) using SPSS (version 22.0.0.2, IBM, New York).

To determine the local observer delineation variation per specific area of the (i)GTV/(i)CTV, we calculated for each patient the median surface, i.e., the surface of the volume that was included by at least 50% of the observers [21]. The median surface was sampled with approximately equidistant points at ~0.5 mm distance. For each point on the median surface, the perpendicular distance to each delineated (i)GTV/(i)CTV was measured. When a delineated surface was not within 2 cm perpendicular to a point on the median surface, the closest distance from that delineated surface to the reference point on the median surface was used instead. For each point on the median surface, the local observer variation was calculated, defined as the SD of the obtained distances at that point (local SD). Per patient, the overall observer variation (overall SD) was calculated. The overall SD was defined as the root-mean-square of the local SDs. Similar as for the CI_gen_, the overall SD was repetitively calculated in a leave-one-out procedure and the range was reported.

The answers to the questionnaire were plotted in a scatterplot using GraphPad Prism and the ratings of the difficulty of the delineation between both stages were compared.

## Results

Eight observers from six different institutions submitted all GTV and iGTV delineations. The analyses of the delineations reported in this results section were performed on the (i)GTV. The results from the (i)CTV are presented in Additional file 2.

### Delineations

**Fig. 1:**
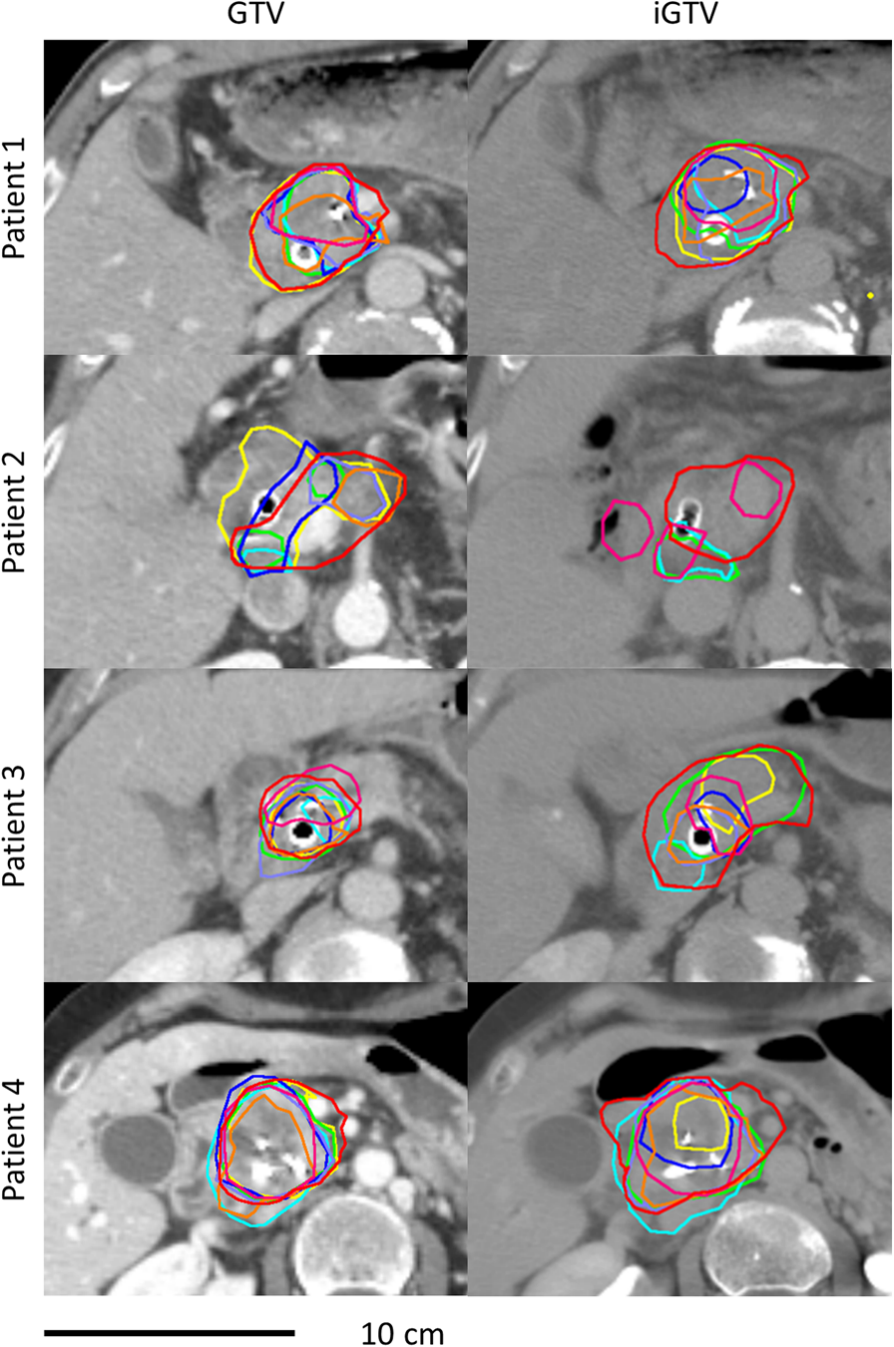
For the four patients, delineations of GTV projected onto an axial 3DCT slice (*left*) and iGTV projected onto an axial 4DCT Ave-IP slice (*right*) for all eight observers. Colors are related to observers and are similar for Figs.2, 3 and 5

**Fig. 2:**
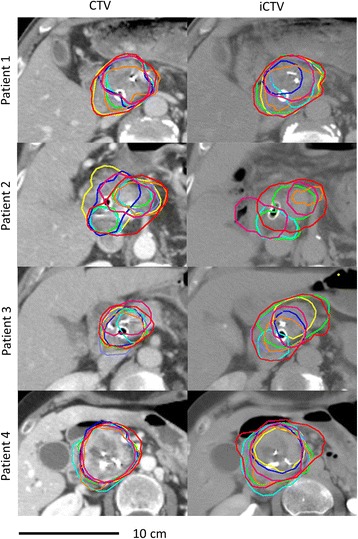
For the four patients, the expansion of the CTV projected onto an axial 3DCT slice (*left*) and iCTV projected onto an axial 4DCT Ave-IP slice (*right*) for all eight observers. Colors are related to observers and are similar for Figs.1, 3 and 5

**Fig. 3:**
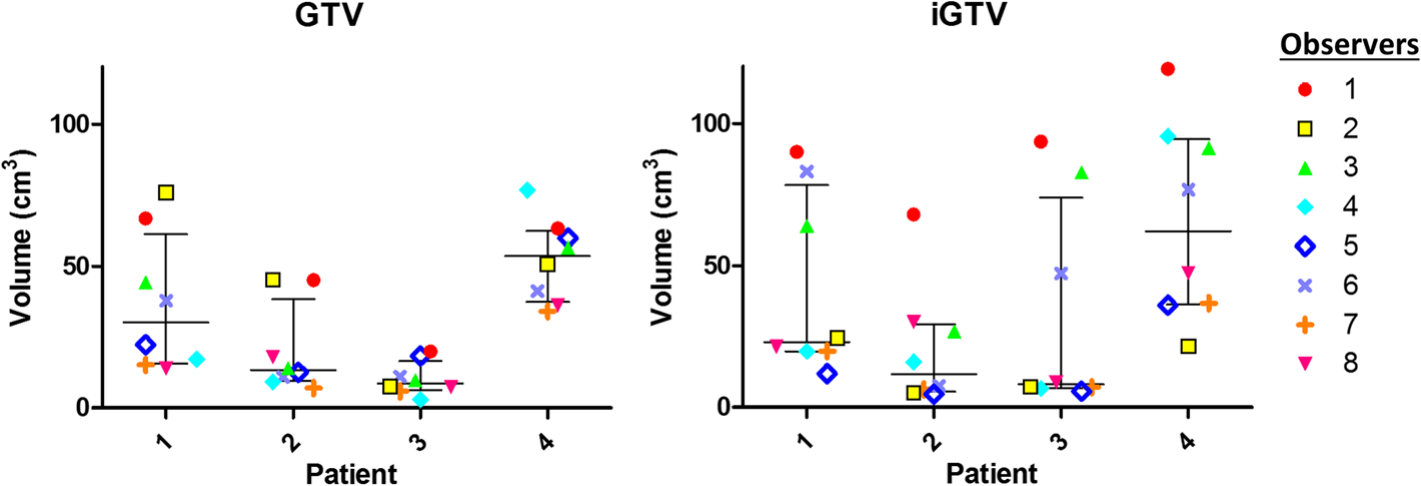
Scatterplots of GTV (*left*) and iGTV (*right*) of all four patients with the median and 25th and 75th percentile represented by the horizontal lines. Colors are related to observers and are similar for Figs.1, 2 and 5

Visual inspection of the delineations revealed considerable interobserver variations (Figs. 1 and 2). The ratio of the largest to the smallest delineated GTV and iGTV was 6.8 and 16.5, respectively, both in patient 3. The iGTV volumes were significantly larger than the GTV volumes by 34% (*P = 0.036*). However, for two observers, the delineated iGTV was smaller than the delineated GTV in all four patients (observers 2 and 5; Fig. 3) and for patient 2 two additional observers (6 and 7) also delineated a smaller iGTV than GTV. Observer 7 reported that his/her iGTV was not based on the ten separate respiratory phases of the 4DCT, due to poor image quality. As the iGTV was delineated on the Ave-IP of the 4DCT, the iGTV still contained 4DCT information. The CI_gen_ was larger for the GTV (mean CI_gen_ =0.37) than for the iGTV (mean CI_gen_ =0.27) for all four patients, indicating a better overlap of volumes in 3D delineation than in 4D delineation (Table 1).

**Table 1:** The average delineated target volumes, overall SDs and CI_gen_ for all 4 patients

Patient		GTV (range^a^)	iGTV (range^a^)
1	Average volume (cm^3^)	36.71 (14.02–75.87)	41.80 (11.85–89.99)
	Overall SD (cm)	0.70 (0.47–0.72)	0.71 (0.60–0.72)
	CI_gen_	0.34 (0.31–0.37)	0.29 (0.26–0.31)
2	Average volume (cm^3^)	20.26 (7.06–45.21)	20.57 (4.67–67.86)
	Overall SD (cm)	0.84 (0.70–0.88)	0.90 (0.37–0.90)
	CI_gen_	0.22 (0.20–0.27)	0.20 (0.17–0.27)
3	Average volume (cm^3^)	10.36 (2.91–19.92)	32.38 (5.67–93.58)
	Overall SD (cm)	0.48 (0.42–0.51)	0.89 (0.77–0.94)
	CI_gen_	0.34 (0.30–0.37)	0.16 (0.12–0.19)
4	Average volume (cm^3^)	52.32 (34.18–76.72)	65.52 (21.48–119.09)
	Overall SD (cm)	0.43 (0.38–0.44)	0.68 (0.58–0.70)
	CI_gen_	0.59 (0.57–0.62)	0.45 (0.42–0.50)
Overall for all patients	Average volume (cm^3^)	29.91	40.07^c^ *(P = 0.036)*
	Overall SD (cm)^b^	0.63	0.80
	CI_gen_	0.37	0.27

*Abbreviations: GTV* gross tumor volume, *iGTV* internal gross tumor volume, *SD* standard deviation, *CI*_*gen*_ generalized conformity index

^a^Range over eight delineations (average volume) or over results of the leave-one-out analysis (overall SD and CI_gen_)

^b^Note that this overall SD was calculated as the root-mean-square of the four overall SDs from the four patients

^c^Two-sided Wilcoxon signed-rank test

### Local observer variation

The local observer variation (local SD) reflects the variation locally projected on the (i)GTV.

**Fig. 4:**
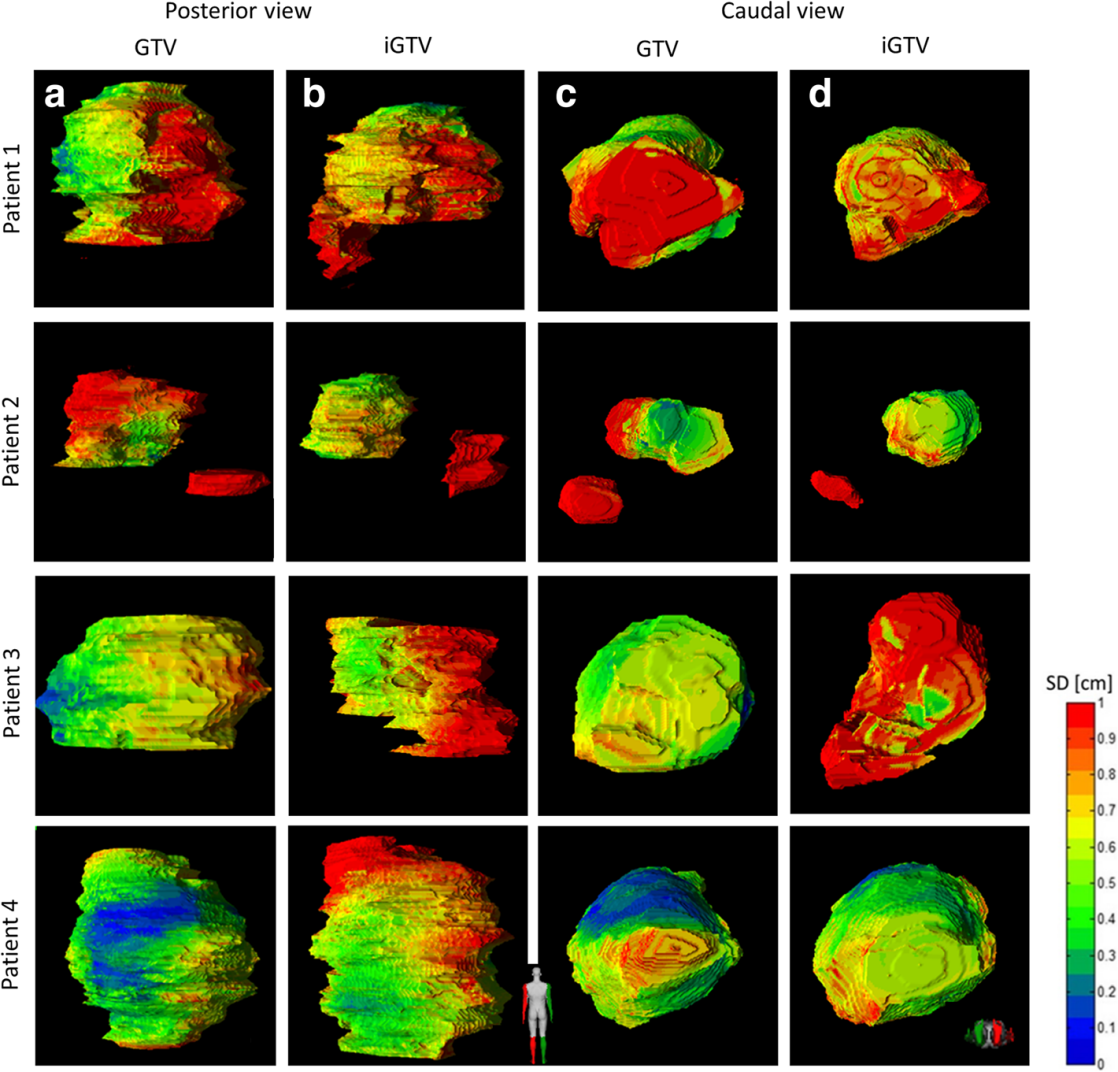
For the four patients, the local observer variation in color expressed in local SD (centimeters) of the delineations of GTV in posterior (**a**) and caudal view (**c**) and the iGTV in posterior (**b**) and caudal view (**d**) projected onto the median surface. Red indicates a local SD ≥ 0.94 cm

There was a large local SD at the laterodorsal borders of the GTV and iGTV of patients 1–3 (Figs. 1 and 4a–b), reflecting the location of the biliary stent. Some observers did, and some did not include the biliary stent in the GTV/iGTV. The biliary stent was included most often in patient 3: by six observers in the GTV and by four observers in the iGTV (Additional file 3). Especially in patient 2, large local variation was seen. The suspicious pathologically enlarged lymph node in the portocaval space was incorporated in the GTV by five (observers 1-5) and in the iGTV by four (observers 1,3,4 and 8) observers. The suspicious pathologically enlarged lymph node along the common hepatic artery was included in the GTV by three (observers 2–4) and in the iGTV by two (observers 3 and 4) observers (Additional file 3). Also for patient 2, only observer 2 included all the misplaced fiducial markers in the GTV and only observer 1 included all the fiducial markers in the iGTV. For all patients, there was some variation in including the fiducial markers in the delineated volume (Additional file 3). Finally, the caudal side of tumors had larger local SDs than the other areas of the tumors (Fig. 4a–d).

### Overall observer variation

The overall observer variation, represented by the overall SDs of the (i)GTV, was smaller for the GTV delineations (SD = 0.63 cm) compared with the iGTV delineations (SD = 0.80 cm) for all four patients (Table 1). Due to the observed discrepancy in including the suspicious pathologically enlarged lymph nodes in patient 2, we recalculated the overall SD while excluding the portocaval lymph node; the overall SD decreased from 0.84 to 0.72 cm for the GTV and from 0.90 to 0.49 cm for the iGTV.

### Questionnaire

**Fig. 5:**
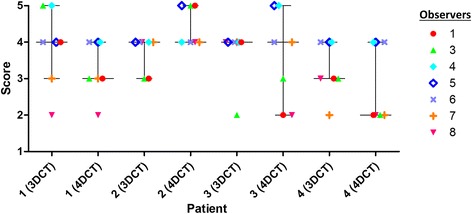
Scatterplot of the delineation difficulty rating by seven observers (observer 2 missing), showing the range, the median, 25^th^, 75^th^ percentile for eight delineations. Rating varied between 1 = very easy; 2 = easy; 3 = moderate; 4 = difficult; 5 = very difficult. Colors are related to observers and are similar for Figs.1, 2 and 3

Seven observers filled out the structured part of the questionnaire; eight observers the open questions. With a mean score of 3.6 for the difficulty of the delineations in both the 3DCT-stage and 4DCT-stage (Fig. 5), the observers did not consider the iGTV (4DCT) delineation more difficult than the GTV (3DCT) delineation. Of the eight observers that filled out the open questions, one radiation oncologist only just started to treat patients with pancreatic cancer. The remaining seven observers treat on average three to four pancreatic cancer patients per year at their institution (range 1–7.5) and on average they had 5.4 years of experience in delineating pancreatic tumors (range 2–12.5). On average, the observers treated one patient (range 0–4) with pancreatic cancer within the PREOPANC trial. Two observers mentioned in the remarks section that the long interval between the diagnostic scan and planning CT scan (average six weeks) made interpretation more challenging.

## Discussion

This multi-institutional delineation study is the first to quantify the interobserver variation on both 3DCT and 4DCT. Also, contrary to earlier studies, this study is performed with more than two patients with (borderline) resectable pancreatic cancer. A considerable variation among observers was observed in both GTV (3DCT) and iGTV (4DCT) delineations. The ratio of the largest to the smallest delineated volume was far larger for iGTV than for GTV, with significantly larger average volumes for the iGTV. Furthermore, the GTV delineations had larger CI_gen_ and smaller overall SDs in all patients compared to the iGTV. The largest variation in delineation was seen close to biliary stents and suspicious pathologically enlarged lymph nodes. Previous studies in pancreatic cancer also showed a large interobserver variation on 3DCT with a comparable ratio of largest to smallest GTV of 3–9 [14–16]. The observed interobserver variation is large compared to studies performed in several other organs such as breast, larynx, and rectal cancer, which reported a CI of 0.6–0.82 [9, 22, 23].

The average iGTV volumes were significantly larger than the GTV volumes by 34%. This is similar compared to previous studies in pancreatic cancer, where the iGTV was 25–27.6% larger than the GTV [2, 24]. As the iGTV should incorporate the GTV in all respiratory phases, this result can be expected. However, unexpectedly, in several cases in our study, observers delineated a smaller iGTV than GTV. This may be a result of a large intraobserver variation, which was not specifically assessed in this study. Alternatively, it could be a result of a difference in image quality between the 3DCT and 4DCT images. It is known that inaccuracies in delineation of the tumor may be due to poorly defined tumor edges on the planning CT images [1, 2, 14–16].

The 4DCT delineations had a larger interobserver variation than the 3D delineations, as reflected in the larger overall SDs of the iGTV compared to the GTV in all four patients and the smaller CI_gen_. This may be the result of poor visibility of the tumor on the various respiratory phases of the 4DCT. Poor visibility can lead to bigger uncertainty and thus larger target volumes and variation in the delineation. The larger interobserver variation on 4DCT counteract the advantage of the ITV concept: accounting for the respiratory motion. Other delineation approaches with improved contrast between tumor and surrounding tissue to define the tumor borders and including the respiration motion should be investigated such as midventilation and particularly the midposition approach. Previous research showed that a midventilation approach results in significant PTV reduction and significant dose reductions to OARs compared to the iGTV approach, although the delineation process had not been investigated yet [6].

The largest local variation was seen at the laterodorsal side of the (i)GTV, corresponding to the location of the biliary stent. Some observers included the stent in the (i)GTV, whereas others excluded the stent. Also, some observers included the stent only in the GTV but not in the iGTV. In the literature, there is no guideline prescribing to include or exclude the biliary stent in the (i)GTV and none was given in the protocol instructions of the PREOPANC trial. The caudal side of the (i)GTV also showed large variations in delineations, similar to a previous study of Caravatta et al. [14].

Also, large variations in the delineation of the suspicious pathologically enlarged lymph nodes around the tumor were seen. The protocol prescribes to include all neighboring suspicious pathological lymph nodes. The reason for the large local variation that was found around suspicious pathologically enlarged lymph nodes could be due to misinterpretation or ambiguity of protocol instructions, or poor compliance with the protocol instructions. This could also result in the wide range of the separate delineations, with ratios of the largest to the smallest GTV and iGTV of 6.8 and 16.5. For the GTV these ratios are similar compared to previous studies [14–16], for the iGTV there is no data to compare. To increase interobserver agreement, consensus on the delineation of pancreatic tumors, pathologically enlarged lymph nodes, and biliary stents should be achieved among radiation oncologists. The proposed guidelines of the high risk nodal areas and CTV delineation described by Carvatta et al. might be used to reduce delineation variation in elective CTVs [13, 14]. The lack of guidelines concerning the GTV margin could be an important cause of increased variation of the boost CTV compared to the elective CTV [14]. Also in the postoperative setting, guidelines serve to develop appropriate radiation fields in the setting of very difficult anatomy in the postoperative setting and to ensure that areas at risk are included in the field while organs at risk are spared [12]. Previous research in other organs showed that national consensus guidelines and a delineation atlas may result in reduction of the interobserver delineation variation [10, 25]. Especially for a clinical trial, improvement of interobserver agreement is important. The study of Abrams et al. showed that failure to adhere to specified radiation guidelines was associated with inferior survival [26].

To optimize tumor visibility, the repetition of the diagnostic scan in treatment position after stenting and placement of the fiducial markers may be a step forward. For the patients in our study, registration between the diagnostic CT and the planning CT was not performed because of a different position of the patient and a different anatomy as a result of the placement of the biliary stents and fiducial markers between both scans. Image registration between a diagnostic scan and planning CT scan may improve accuracy in target delineation and reduce interobserver variation as seen for other tumor sites [27–29].

It is well known that pancreatic tumors are difficult to distinguish from normal pancreas tissue on diagnostic CT scans [2, 30, 31]. Therefore, exploitation of other imaging modalities, such as MRI and PET-CT may be a step forward to reduce the variation in delineation of pancreatic tumors. Indeed, other studies have shown that additional imaging, such as MRI and PET-CT, may be helpful in the delineation of pancreatic tumors [32, 33].

### Limitations

Delineations were only performed once, and we could not investigate the intraobserver variation. Furthermore, we had a limited number of responding observers, and only a limited number of patients were included. Also, the observers had little experience in the delineation of pancreatic tumors, due to the small number of pancreatic cancer patients eligible for radiotherapy. However, this is typical for many radiation oncologists and hence the found observer variations should be representative for such radiation oncologists.

The time interval between diagnostic CT and planning CT scan was on average six weeks and the patients were not scanned in treatment position; therefore, anatomical changes (including placement of the biliary stent) occurred between both scans and scans were not registered. This made it challenging to delineate the (i)GTV. However, this is a typical situation in clinical practice in many hospitals since the diagnostic CT is obtained before histological diagnosis, while therapeutic measures such as stenting are performed after the diagnostic CT scan. The fiducial markers in patient 2 were mistakenly not placed inside the tumor, which may have put some observers on the wrong track and contributed to the large interobserver variation seen in this patient.

## Conclusion

This study showed a considerable interobserver variation in delineation of pancreatic tumors, larger for 4DCT than for 3DCT delineation. The local variation was largest around the biliary stent and suspicious pathologically enlarged lymph nodes. In the future, better guidelines and the addition of other imaging modalities, such as PET or MRI may help decrease observer variation.

## Additional files

Additional file 1: Questionnaire. (PDF 258 kb)

Additional file 2: Data of CTV and iCTV. (PDF 464 kb)

Additional file 3: Inclusion of suspicious pathological lymph nodes, stents and fiducials in the delineations. (PDF 279 kb)

## Abbreviations

AMC: Academic medical center
Ave-IP: Average intensity projection
CI_gen_: Generalized conformity index
CT: Computed tomography
CTV: Clinical target volume
GTV: Gross tumor volume
iCTV: Internal clinical target volume
iGTV: Internal gross tumor volume
ITV: Internal target volume
MIP: Maximum intensity projection
OAR: Organ at risk
PTV: Planning target volume
SD: Standard deviation
