# Supplementary material for: Considerable interobserver variation in delineation of pancreatic cancer on 3DCT and 4DCT: a multi-institutional study
Source: Radiat Oncol. 2017 Mar 23;12:58. doi: 10.1186/s13014-017-0777-0 (PMC5364627; doi:10.1186/s13014-017-0777-0)
Supplement: Supplementary file 1 — Questionnaire. (PDF 258 kb) [file 13014_2017_777_MOESM1_ESM.pdf]

Additional file 1

Questionnaire

1. *How did you experience the delineation of the pancreatic tumor of patient 1 on 3DCT?*
    a. Very Easy
    b. Easy
    c. Moderate
    d. Difficult
    e. Very difficult
2. *How did you experience the delineation of the pancreatic tumor of patient 2 on 3DCT?*
    a. Very Easy
    b. Easy
    c. Moderate
    d. Difficult
    e. Very difficult
3. *How did you experience the delineation of the pancreatic tumor of patient 3 on 3DCT?*
    a. Very Easy
    b. Easy
    c. Moderate
    d. Difficult
    e. Very difficult
4. *How did you experience the delineation of the pancreatic tumor of patient 4 on 3DCT?*
    a. Very Easy
    b. Easy
    c. Moderate
    d. Difficult
    e. Very difficult
5. *How did you experience the delineation of the pancreatic tumor of patient 5 on 4DCT?*
    a. Very Easy
    b. Easy
    c. Moderate
    d. Difficult
    e. Very difficult
6. *How did you experience the delineation of the pancreatic tumor of patient 6 on 4DCT?*
    a. Very Easy
    b. Easy
    c. Moderate
    d. Difficult
    e. Very difficult
7. *How did you experience the delineation of the pancreatic tumor of patient 7*

on 4DCT?<br>a. Very Easy<br>b. Easy<br>c. Moderate<br>d. Difficult<br>e. Very difficult
8. *How did you experience the delineation of the pancreatic tumor of patient 8 on 4DCT?*<br>a. Very Easy<br>b. Easy<br>c. Moderate<br>d. Difficult<br>e. Very difficult
9. *How many years' experience do you have in delineation pancreatic tumors for radiotherapy?*
10. *How many patients with pancreatic tumors do you treat per year?*
11. *Have you treated patients within the PREOPANC study?*
