# Supplementary material for: Considerable interobserver variation in delineation of pancreatic cancer on 3DCT and 4DCT: a multi-institutional study
Source: Radiat Oncol. 2017 Mar 23;12:58. doi: 10.1186/s13014-017-0777-0 (PMC5364627; doi:10.1186/s13014-017-0777-0)
Supplement: Supplementary file 2 — Data of CTV and iCTV. (PDF 464 kb) [file 13014_2017_777_MOESM2_ESM.pdf]

Additional file 2

Data of CTV and iCTV

Table e1. The volumes, overall standard deviations and conformity indexes of all 4 patients

**Patient**		**CTV (range*)**	**iCTV (range*)**
**1**	Average volume (cm^3)	79.21 (36.04–145.96)	89.15 (31.70–165.48)
	Overall SD (cm)	0.75 (0.60–0.77)	0.82 (0.69–0.85)
	CI_{gen}	0.43 (0.41–0.46)	0.38 (0.35–0.41)
**2**	Average volume (cm^3)	52.99 (24.97–106.90)	53.05 (16.59–131.79)
	Overall SD (cm)	0.90 (0.80–0.95)	1.05 (0.39–1.05)
	CI_{gen}	0.32 (0.29–0.37)	0.30 (0.27–0.34)
**3**	Average volume (cm^3)	28.66 (11.85–46.76)	68.26 (19.36–169.21)
	Overall SD (cm)	0.52 (0.45–0.55)	0.99 (0.88–1.04)
	CI_{gen}	0.46 (0.43–0.50)	0.23 (0.19–0.26)
**4**	Average volume (cm^3)	103.86 (75.13–147.46)	126.75 (49.2–204.04)
	Overall SD (cm)	0.44 (0.39–0.46)	0.72 (0.62–0.74)
	CI_{gen}	0.65 (0.63–0.67)	0.50 (0.47–0.54)
**Overall for all patients**	Average volume (cm^3)	66.18	84.30‡ (*P=0.045)*
	Overall SD (cm)§	0.68	0.91
	CI_{gen}	0.46	0.35

Abbreviations: CTV, clinical target volume; iCTV, internal clinical target volume; SD, standard deviation; CI_{gen}, generalized conformity index.

* Range over 8 delineation (average volume) or results of leave-one-out analysis (overall SD and CI_{gen}).

† Note that the overall SD was calculated as the root-mean-square of the four SDs from the four patients

‡Two-sided Wilcoxon signed-rank test

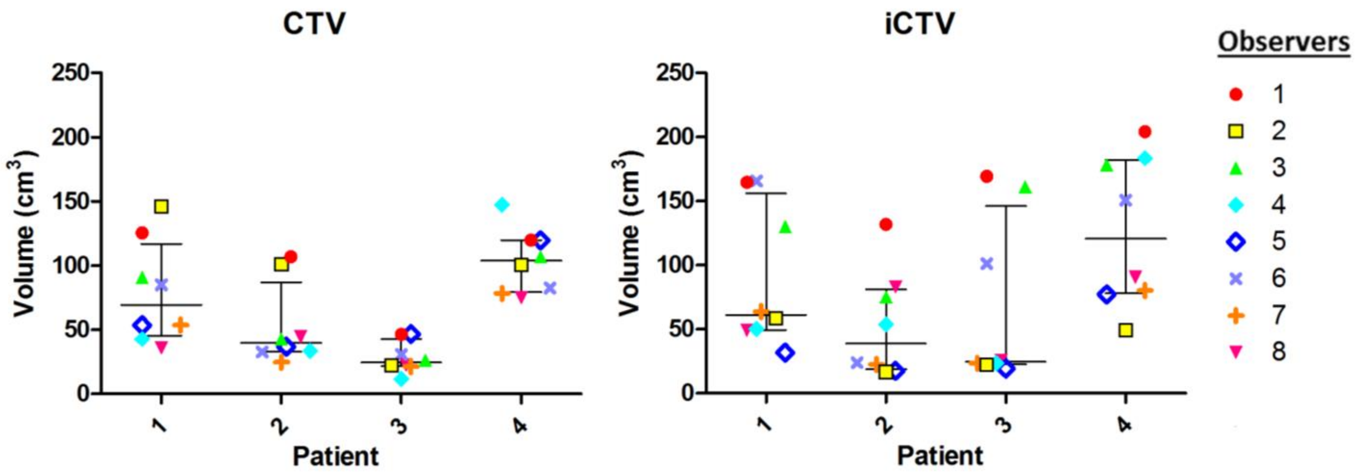

Fig. e1: Scatterplots of CTV (left) and iCTV (right) of all four patients with the median, 25th and 75th percentile represented by the horizontal lines. Colors are related to observers and are similar for Fig. 3 in the paper.
