# Supplementary material for: Considerable interobserver variation in delineation of pancreatic cancer on 3DCT and 4DCT: a multi-institutional study
Source: Radiat Oncol. 2017 Mar 23;12:58. doi: 10.1186/s13014-017-0777-0 (PMC5364627; doi:10.1186/s13014-017-0777-0)
Supplement: Supplementary file 3 — Inclusion of suspicious pathological lymph nodes, stents and fiducials in the delineations. (PDF 279 kb) [file 13014_2017_777_MOESM3_ESM.pdf]

Additional file 3

Suspicious pathological lymph nodes, stents, and fiducials

Table e2. Number of observers who included the suspicious pathological lymph nodes in the (i)GTV according the diagnostic CT report.

Patient	Location suspicious pathological lymph node	GTV	iGTV
2	Portocaval	5	4
2	Along common hepatic artery	3	2
4	Along tumor, not characterized	2	2

Table e3. Number of observers who included the biliary stent or percutaneous biliary drainage in the (i)GTV at least 50% of the stent in at least three slices

Patient	GTV-CT	iGTV-CT
1	3	5
2	2	1
3	6	4
4	8	7

Table e4. The number of (i)GTV including the fiducial over the number of fiducials multiplied by number of observers (3×8=24). Partly included fiducials were counted as delineated.

Patient	GTV-CT	iGTV-CT
1	24/24 (100 %)	23/24 (100 %)
2*	5/24 (21 %)	4/24 (17 %)
3	22/24 (92 %)	16/24 (67 %)
4	24/24 (100 %)	21/24 (88 %)

* In patient 2, the fiducials were mistakenly not implanted in the tumor
